# Biosynthetic Machinery Involved in Aberrant Glycosylation: Promising Targets for Developing of Drugs Against Cancer

**DOI:** 10.3389/fonc.2015.00138

**Published:** 2015-06-25

**Authors:** Andréia Vasconcelos-dos-Santos, Isadora A. Oliveira, Miguel Clodomiro Lucena, Natalia Rodrigues Mantuano, Stephen A. Whelan, Wagner Barbosa Dias, Adriane Regina Todeschini

**Affiliations:** ^1^Instituto de Biofísica Carlos Chagas Filho, Universidade Federal do Rio de Janeiro, Rio de Janeiro, Brasil; ^2^Department of Biochemistry, Cardiovascular Proteomics Center, Boston University School of Medicine, Boston, MA, USA

**Keywords:** glycans, glycosyltransferases, inhibitors, cancer, hexosamine biosynthetic pathway, O-linked glycan, N-linked glycan, glycoconjugate

## Abstract

Cancer cells depend on altered metabolism and nutrient uptake to generate and keep the malignant phenotype. The hexosamine biosynthetic pathway is a branch of glucose metabolism that produces UDP-GlcNAc and its derivatives, UDP-GalNAc and CMP-Neu5Ac and donor substrates used in the production of glycoproteins and glycolipids. Growing evidence demonstrates that alteration of the pool of activated substrates might lead to different glycosylation and cell signaling. It is already well established that aberrant glycosylation can modulate tumor growth and malignant transformation in different cancer types. Therefore, biosynthetic machinery involved in the assembly of aberrant glycans are becoming prominent targets for anti-tumor drugs. This review describes three classes of glycosylation, O-GlcNAcylation, N-linked, and mucin type O-linked glycosylation, involved in tumor progression, their biosynthesis and highlights the available inhibitors as potential anti-tumor drugs.

## Introduction

**Figure 1:**
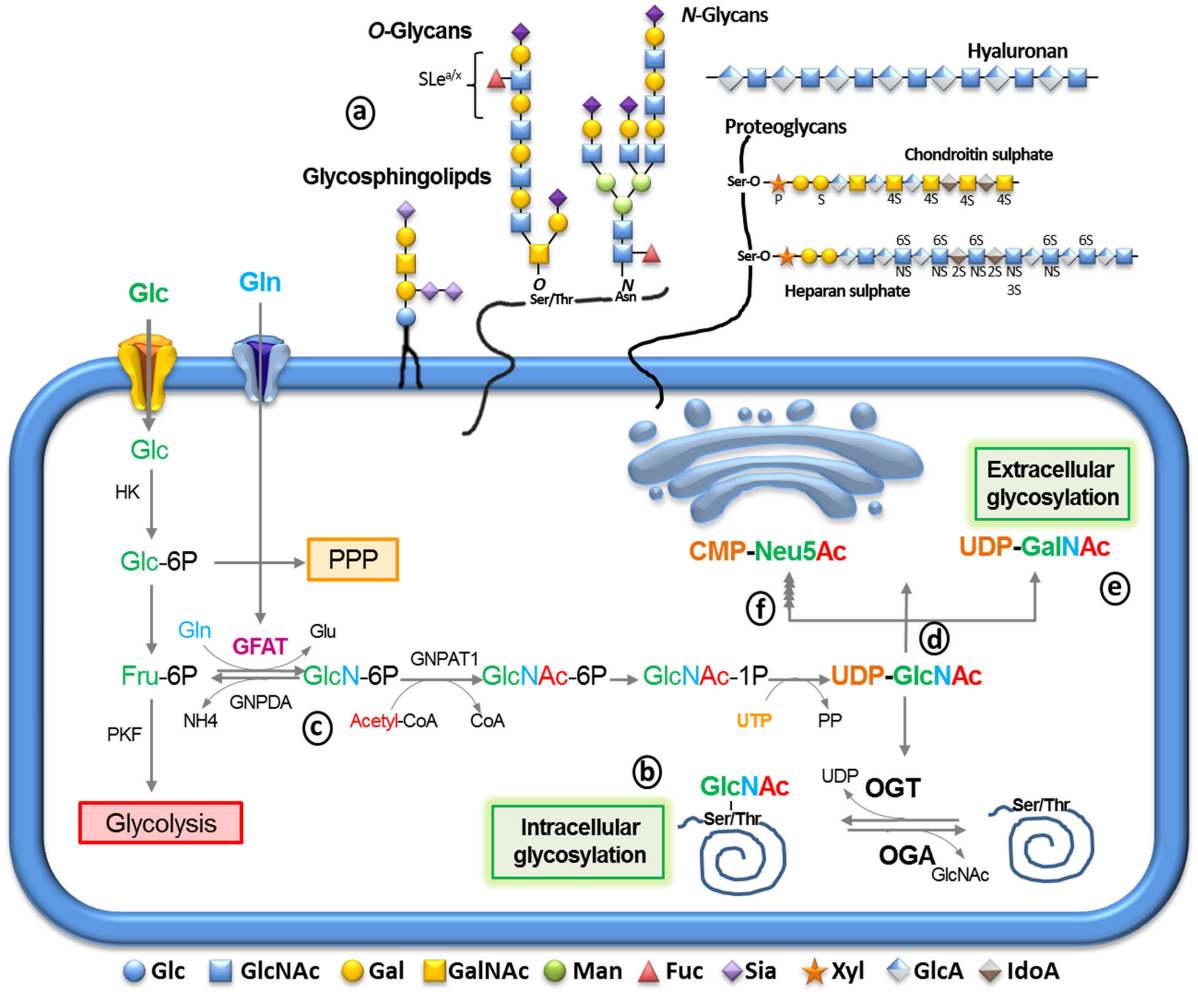
**The synthesis of glycoconjugates from glucose through the hexosamine biosynthetic pathway (HBP)**. After glucose entry into the cell via the glucose transport, it is phosphorylated into glucose-6-phosphate (Glc-6P) by hexokinase (HK), mainly proceeding into glycolysis through conversion into fructose-6­-phosphate (Fru-6P) by Glc-6P isomerase. Alternatively, Glc-6P may be utilized by the pentose phosphate pathway (PPP). Glc-6P can also be diverted to glucosamine-6-phosphate by the rate-limiting enzyme glutamine:fructose-6-phosphate amidotransferase (GFAT) (c). The end product of this pathway, uridinediphosphoglucose-*N*-acetylglucosamine (UDP-GlcNAc) (d) serves to build extracellular glycoconjugates (a), as well as, it is used for the biosynthesis of intracellular O-linked glycoproteins (b) by the enzyme O-GlcNAc transferase. Alternatively, UDP-GlcNAc can undergo epimerization to generate UDP-GalNAc (e) and CMP-Neu5Ac (f) which can be used for the extracellular biosynthesis of glycoproteins and glycolipids (a) UDP-GlcNAc and its derivatives are extremely responsive to variations in cell nutrients as its synthesis depends on products of the metabolism of glucose (green), amino acids (blue), fatty acids (red), and nucleotides (orange). Thus, glycosylation can serve as a reporter for the functional status of multiple pathways and considered a metabolic sensor.

Glycans constitute the most complex and abundant group of molecules in living organisms. Besides playing important roles in energy storage and supply, they often serve as essential biosynthetic precursors or structural elements needed to sustain all forms of life. The complex glycans are frequently attached to proteins, forming glycoproteins and proteoglycans, or to lipids, forming glycosphingolipids and glycosylphosphatidylinositol anchors (Figure 1a). The majority of glycoconjugates are expressed on the cell surface, where they form a thick layer known as glycocalyx. Glycans can also be secreted to the extracellular medium in order to be incorporated into the extracellular matrix (ECM). Such location places glycoconjugates as major players in cell-to-cell interactions and motility. In addition, glycosylation is analogous to phosphorylation in that it can be found on many, cytoplasmic, nuclear and mitochondrial proteins (Figure 1b). It is a dynamic post-translational modification (PTM) and it regulates many cellular functions as well (1).

Glycoconjugates participate in many key biological processes including cellular adhesion, migration, growth, differentiation, signal transduction, receptor activation, immune response modulation, quality control of protein folding, and host–pathogen interactions (2–4).

Glycans play several roles in different steps of tumor progression regulating tumor proliferation, invasion, metastasis, and angiogenesis (5). Therefore, aberrant glycosylation exhibits prominent candidates for cancer biomarkers, and their biosynthetic machinery have become targets for designing and synthesizing anti-tumors drugs.

Glycan structures do not depend only on genes, but also on the activities of glycosyltransferases and glycosidases and the availability of the donor substrates at the needed location. The donor substrates are derived from extracellular glucose (Glc) and from intracellular degradation of glycoconjugates in lysosomes, through the action of glycosidases and others enzymes as epimerases. Tumor cells have altered Glc metabolism, producing ATP primarily through glycolysis even under normoxic condition, thereby upregulating the Glc uptake approximately 10 times more than adjacent normal tissue (6) in order to sustain a highly demanding metabolism. This metabolic shift was termed “Warburg effect” and is critical for supporting the malignant phenotype (6). The high rate of glycolytic flux is a central metabolic hallmark of tumors and cancer cells support this rate by increasing the expression of Glc transporters (Glut) (7). This phenomenon of elevated Glc uptake has been clinically exploited to detect tumor cells by positron emission tomography (PET) scan (8). In addition, a stable Glc analog 2-deoxi-d-glucose (2-DG) has been suggested as a tumor therapeutic drug (9, 10). Various small molecule inhibitors of the glycolytic pathway have been used effectively in the past to halt the progression of cancer (11–13). The 2DG is a well-known glycolytic inhibitor, which inhibits the key glycolytic enzyme hexokinase. Recently, Muley et al. (10) evaluated the additional cellular effects of 2DG, apart from inhibiting glycolysis. Their findings indicate that 2DG increases the expression of p21 and p53 in colorectal cancer cell lines leading to cell cycle arrest at the G0/G1 phase.

## Hexosamine Biosynthetic Pathway

Upon entering cells, Glc is rapidly converted to glucose-6-phosphate (Glc-6P) by hexokinase (Figure 1) and then to fructose-6-phosphate (Fru-6P) by glucose-6-phosphate isomerase. Despite the majority of Fru-6P being metabolized by phosphofructokinase (PFK) entering in to glycolysis, approximately 2–5% of Glc influx is directed by the hexosamine biosynthetic pathway (HBP) (14). The first and rate limiting step of the HBP is catalyzed by glutamine, fructose-6-phosphate amidotransferase (GFAT), which converts Fru-6P to glucosamine-6-phosphate (GlcN-6P) using glutamine (Gln) as an amine donor (Figure 1c).

GlcN-6P is further metabolized to uridine-5'-diphosphate-*N*-acetylglucosamine (UDP-GlcNAc) that serves as a major substrate for several kinds of glycosylation including O-linked *N*-acetylglucosamine (*O*-GlcNAc), *O*-glycans, *N*-glycans, glycosaminoglycans, and glycolipids (Figure 1d). UDP-GlcNAc can be epimerized to uridine-5′-diphospho-*N*-acetylgalactosamine (UDP-GalNAc; Figure 1e) or further metabolized to generate cytidine-5´-monophosphate-5-*N*-acetylneuraminic acid (CMP-Neu5Ac; Figure 1f). UDP-GlcNAc and its derivatives are considered sensors of the metabolic status of the cell, as it requires components of all four major classes of macromolecules: Glc, Gln, acetyl-coenzyme-A, and the nucleotide UDP. Gln is a key nutrient for tumor cells, being a major source of nitrogen and energy in rapidly dividing cells (15). Although the cause of increased flux through the HBP is not clear in tumor cells, it is likely to occur as a result of increased Glc and Gln uptake. To support this hypothesis, Itkonen et al. recently showed that several HBP genes were overexpressed in human prostate cancers (16). Thus, the link between altered metabolism and the up-regulation of glycosylation through the HBP provides a mechanism for cancer cells to sense and respond to a variety of environmental conditions.

How the HBP induces the malignancy process is not completely understood yet. One hypothesis is that the HBP exerts its effects by transforming growth factor-β (TGF-β) secretion. Many manuscripts have described that elevated Glc levels induce TGF-β production by different cell lines (17, 18). TGF-β is a known potent inductor of epithelial mesenchymal transition (EMT). The EMT involves a striking decline in epithelial markers, such as E-cadherin, ocludins, claudins, cytokeratin, and consequently cell polarity, accompanied by enhanced expression of mesenchymal markers, such as *N*-cadherin, vimentin, and fibronectin (FN), culminating in cell morphology alteration and increased cell motility (19). Besides, recent studies bring to light the involvement of a key *O*-glycosylation in the IIICS, a variant splicing domain of human FN, forming the oncofetal fibronectin (onfFN) during the EMT process (20). The importance of glycosylation in this process was supported by data showing that ppGalNAc-T6 knockdown inhibits onfFN biosynthesis and EMT in human prostate epithelial cells (20). In addition, a recent manuscript indicated that high Glc or GFAT2 overexpression induces EMT, onfFN production and increased ppGalNAc-T6 mRNA levels in human alveolar epithelial adenocarcinoma cells. Those factors imply that metabolite availability to the HBP exerts control over gene expression and modulates cell surface glycosylation, suggesting that changes in Glc uptake alters epithelial cell communication with neighboring cells and the ECM, which results in loss of tissue organization and contributes to tumor formation and progression (21). Thus, it is reasonable to think that glycan structures are changed by the metabolic status of the cells, and the aberrant glycosylation observed in tumors is a consequence of altered expression of glycosyltransferases combined with substrates availability. Therefore, the metabolic pathways, especially the HBP, can be directly implicated in alterations observed in *O*-GlcNAcylation (1), *N*-glycans (22), and *O*-glycans (21) in cancer cells.

## O-Linked *N*-Acetylglucosamine

The *O*-GlcNAc PTM is characterized by the linkage of a β-*N*-acetylglucosamine moiety to the hydroxyl group of threonine (Thr) or serine (Ser) residues found in nuclear, cytoplasmic, and mitochondrial proteins (1). The addition of *O*-GlcNAc to proteins is catalyzed by *O*-GlcNAc transferase (OGT), and its removal is catalyzed by *O*-GlcNAcase (OGA). Deletion of OGT is lethal in mice at embryonic and single-cell level, highlighting the importance of *O*-GlcNAcylation in regulating basic cellular events (23). Aberrant *O*-GlcNAcylation has been linked to major diseases, including cancer, diabetes, and Alzheimer’s disease (24, 25). This dynamic glycosylation is analogous to phosphorylation and more than 1000 proteins have been described to be *O*-GlcNAcylated to date (26). The relationship between phosphorylation and *O*-GlcNAcylation has proved more complex than initially thought, since their function is not limited to site occupancy alone, but both PTMs can modulate each other at the same site or adjacent sites (27). Our group has also shown that *O*-GlcNAc can modulate tyrosine (Tyr) phosphorylation, indicating that the interplay between these PTMs at the substrate level is not limited to Ser and Thr residues (28). Growing evidence suggests that *O*-GlcNAcylation and phosphorylation not only compete for substrates (at the same or proximal sites), but also that *O*-GlcNAcylation regulates kinases and/or phosphatases. In one example, we showed that *O*-GlcNAcylation directly regulates the kinase activity of calcium/calmodulin-dependent protein kinase type IV (CaMKIV) toward cAMP response element-binding protein (CREB) (29). In addition, we recently showed that 39% of the kinases of the functional protein array are *O*-GlcNAcylated *in vitro* by recombinant OGT. Interestingly, the majority of identified kinases play a role in cancer (30).

Like phosphorylation, *O*-GlcNAcylation can modulate protein function, turnover, interactions, subcellular localization, enzyme activity, DNA affinity, and transcription activity (27). Several transcription factors involved in cancer biology, such as p53, c-Myc, NF-kB, and Sp1 are modified by *O*-GlcNAc (31). Over 60 papers were published in the past 3 years describing the relationship between *O*-GlcNAc and cancer, with a substantial portion of them related to the increase of *O*-GlcNAc and OGT in several types of tumors (32, 33). Increased protein *O*-GlcNAcylation and changes in OGT expression have been described in breast cancer, lung cancer, prostate cancer, pancreatic cancer, and colorectal cancer (16, 34–38). In addition, OGT silencing inhibits tumor growth in different models including breast cancer, prostate cancer, and pancreatic cancer (34, 37, 38), indicating that *O*-GlcNAcylation is important for tumorigenesis and suggesting that OGT represents a novel therapeutic target for these types of cancers (39). Interestingly, a low expression of OGA is suggested as a prognostic marker for hepatocellular carcinoma tumor recurrence (40). Another study suggests that the urinary content of OGT and OGA may be useful for bladder cancer diagnostics (41).

Recently, Hsieh-Wilson’s group showed that PFK is *O*-GlcNAcylated at Ser529 during hypoxia (42). This glycosylation inhibited the PFK activity and redirected the Glc flux through the pentose phosphate pathway (PPP), increasing the reducing power of the cell by the production of nicotinamide adenine dinucleotide phosphate (NADPH) and glutathione (GSH). Such a shift in metabolic flux confers a selective growth advantage for cancer cells, since blocking the glycosylation of PFK at Ser529 reduced cancer cell proliferation *in vitro* and impaired tumor formation *in vivo* (42). Thus, blocking PFK1 glycosylation would provide a new strategy to combat cancer.

Despite evidence linking aberrant *O*-GlcNAcylation to cancer (1, 33, 43), only a few studies show how *O*-GlcNAc participates in the molecular mechanism involved in EMT. *O*-GlcNAcylation at serine 112 of Snail, the repressor of E-cadherin, blocks its phosphorylation by GSK3β and protects Snail from ubiquitylation and degradation. Hyperglycemic condition enhances *O*-GlcNAc modification and initiates EMT by transcriptional suppression of E-cadherin through Snail (44). Moreover, treatment of low metastatic human ovarian cancer cells (OVCAR-3) with the OGA inhibitors Thiamet-G and PUGNAc enhances their migration potential and decreases the expression of E-cadherin, consequently blocking the formation of the E-cadherin/catenin complex reducing intracellular adhesion. Whereas, when a high metastatic ovarian cancer cell lineage (HO-8910PM) are subjected to OGT silencing, the expression of E-cadherin is recovered and their potential migration ability is diminished (45). Taken together, the data demonstrate that *O*-GlcNAc plays an important role in EMT events, cell migration, and gain of malignancy and metastasis, which suggests it could be a potential target for cancer treatment.

Although OGT is a promising target against different types of tumors, OGT is an essential glycosyltransferase that targets specific sites on hundreds of protein substrates, making its inhibition a difficult task. Studies indicate that the specificity of OGT toward different substrates is modulated by transient associations with binding partners (46, 47). Recently, the structure of human OGT and the mechanism of action have been reported (48–50). Despite a series of new advances in structural and mechanistic features of OGT, the existing inhibitors for this enzyme are not as specific and efficient as the OGA inhibitors (51). In addition, whereas almost all OGT inhibitors target the UDP-GlcNAc binding site, they are not as potent as the reaction product UDP, which displays a *K*_d_ of 0.5 μM. Although studies have only been performed in cell culture, we highlight three OGT inhibitors: (i) ST045849 (**1**; Scheme 1 in Supplementary Material) that has been used successfully in the inhibition of prostate cancer cell lines (16); (ii) ST060266 or BZX (**2**; Scheme 1), an irreversible inhibitor of OGT used in different cell types (49); and (iii) 4Ac-5S-GlcNAc, a cell-permeable compound that enters the HBP to be synthesized into UDP-5S-GlcNAc (**3**; Scheme 1), a potent OGT inhibitor (52). Interestingly, a recent OGT bisubstrate inhibitor, presenting an acceptor peptide linked to UDP has emerged as a new scaffold for the development of more specific inhibitors (53).

## N-Linked Glycans

Many cell surface, lysosomal, and secreted proteins are post-translationaly modified by the addition of a β-GlcNAc to the asparagine (Asn) residues (N*-*linked) of the evolutionary conserved “sequon” Asn-X-Ser/Thr, where X is any amino acid except proline. N-linked glycans consist of a conserved pentasaccharide core Manα1-6(Manα1-3)Manβ1-4GlcNacβ1-4GlcNacβ1-Asn trimmed with different sugars, organized in up to five antennae branches. Such variable structures create an array of glycoforms with different physical and biochemical properties that confer functional diversity to the glycoprotein. N-glycosylation affects protein folding, playing a central role in protein quality control within the endoplasmic reticulum (ER), in metastatic potential and the spread of tumors (54).

Changes in the oligosaccharide structure of N-glycans have been described in breast, colon, prostate, lung, renal cell, hepatocellular carcinoma, pancreatic, and gastric cancer (55–67). Most growth factor receptors on the cell surface are N-glycosylated, including epithelial growth factor receptor (EGFR) (68), integrins (69), and TGF β receptor (TGFβR) (70). *N*-glycans are ligands for galectin 1 and 3 (71) and siglecs (72) at the cell surface, forming lattices that enhance the residence time of receptors (73–75). Oncogenesis has been shown to upregulate the expression of *N*-glycans with higher affinity for galectins and to increase the residence time of the receptors (76–79). These studies open the possibility to target surface distribution of growth factor receptors by modulating *N*-glycan branching. Such strategies might be useful in cancer therapy. Interestingly, inhibition of the *N*-glycan biosynthetic pathway is emerging as an exciting target to inhibit cancer progression.

The first steps of *N*-glycan biosynthesis are conserved among eukaryotic cells (80–82). The pathway begins at the cytoplasmic face of the ER membrane with the assembly of a 7-residue oligosaccharide precursor linked to the lipid dolichol-phosphate. Two GlcNAc molecules from the highly energetic donor GlcNAc-PP-dolichol are the first residues incorporated into dolichol-pyrophosphate. The activated monosaccharide donor is synthesized by the GlcNAc phosphotransferase, which transfers the GlcNAc-1-phosphate from UDP-GlcNAc (83). The oligosaccharide is further assembled, step by step, by specific glycosyltransferases that add other five mannoses (Man) to the disaccharide. The dolichol oligosaccharide chain anchored to the cytosolic side is moved into the lumen of the ER. Within this organelle, four Man and three Glc residues are added to the growing glycolipid.

The mature lipid-linked precursor is further transferred as a single block from the dolichol to Asn residues of nascent peptide by the transmembrane oligosaccharyl transferase protein complex (82). In the next step, glucosidases trim two terminal Glc from the glycoprotein Asn-GlcNAc_2_Man_9_Glc_3_, a crucial process for proper protein folding. The final Glc undergoes several cycles of removal and reintroduction while the process of protein folding is assisted by two lectin chaperones, known as calnexin and calreticulin, which bind to terminal Glc. After correct folding, the Glc and Man unities are removed by α-glucosidases and α-mannosidases, respectively.

Inhibition of protein folding in the lumen of the ER results in ER stress (84). ER stress activates various intracellular signaling pathways, known as the unfolded protein response (UPR), which is comprised by the inhibition of general protein translation, the induction of genes such as ER chaperones that increase the protein-folding capacity of the ER, and upregulation of aberrant protein degradation. The severe stress induces apoptosis and elimination of the damaged cell. In recent years, many groups have been seeking to pharmacologically aggravate chronic ER stress in cancer cells in order to enhance apoptosis and achieve tumor cell death.

Inhibition of *N*-glycan biosynthesis can impact protein folding and is emerging as an interesting strategy to reduce receptor tyrosine kinase (EGFR, ERβB2, insulin-like growth factor-I receptor, and Met) maturation and its cell surface expression in multiple cancers, including squamous cell carcinomas, adenocarcinomas of the breast, prostate and pancreas, and malignant gliomas (85–87). Along this line, it has been reported that ER stress induced by tunicamycin (**4**; Scheme 1) influences chemosensitivity of tumor cells to anticancer drugs (88) and to radiotherapy (85, 86, 89, 90) and influences tumor induced angiogenesis (91). Tunicamycin is a mixture of homologous nucleoside antibiotics that competitively inhibits (*K*_i_ 5 × 10^–8^ M) the enzyme GlcNAc phosphotransferase, which prevents the formation of GlcNAc-PP-dolichol (83, 92). Tunicamycin inhibits cell invasion and tumorigenicity both *in vitro* and *in vivo* (93–99). Other antibiotics that inhibit the lipid-linked saccharide pathway are amphomycin (**5**; Scheme 1), tridecaptin (**6**; Scheme 1), flavomycin (**7**; Scheme 1), diumycin (**8**; Scheme 1), and tsushimycin (**9**; Scheme 1) (83, 100).

Inhibition of protein folding can also be achieved by inhibiting glycoprotein-processing enzymes ensuing anti-tumoral activity (101–104). Castanopermine (**10**; Scheme 1) inhibits glucosidase I and leads to altered glycoproteins with Glc3Man7GlcNAc2 structures (105). The recent report of the first structural model of eukaryotic α-glucosidase (106) will improve the design and synthesis of novel enzyme inhibitors, which will hopefully be more effective against cancer.

**Figure 2:**
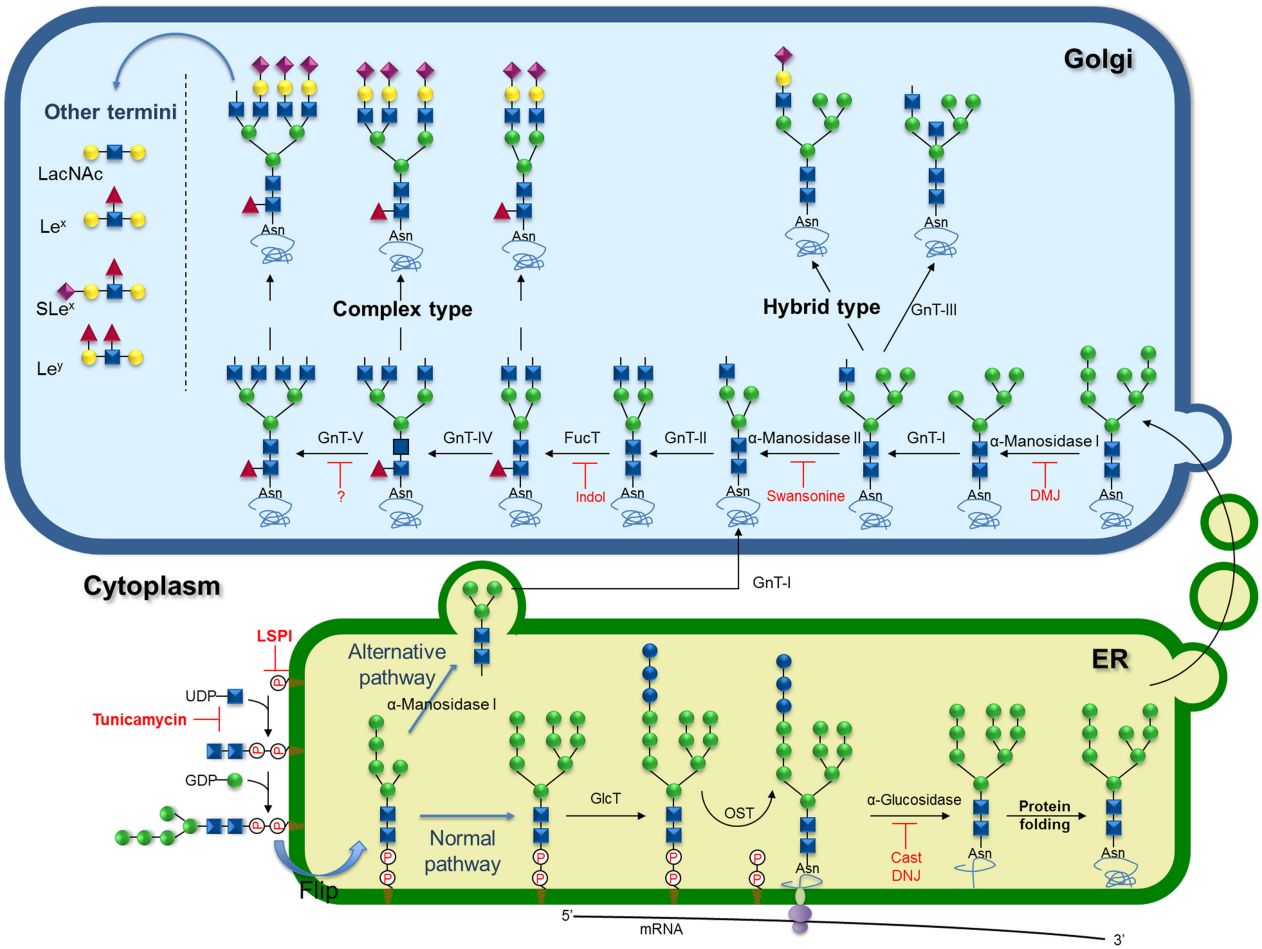
**Schematic representation of biosynthesis and processing of N-linked oligosaccharides showing known inhibitors and key targets for inhibition**. Inhibitors of the lipid-linked saccharide pathway (LSPi), tunicamycin, castanopermine (Cast), 1-deoxynojirimycin (DNJ), 1-deoxymannojirimycin (DMJ), swansonine, and indolizidine (Indol).

The processed glycoprotein, therefore, moves to the Golgi where it is demannosylated by the Golgi α-mannosidase I forming the Man_5_GlcNAc_2_ structure, which is a substrate for the acetylglucosaminyltrasferase-I (GnT-I), a key enzyme in the development of multicellular organisms (107, 108). The addition of the first GlcNAc residue by GnT-I generates the GlcNAcMan_5_GlcNAc_2_, substrate for GnT-III or α-mannosidase II (Figure 2). α-Mannosidase II trims two Man residues from the intermediate to form the core GlcNAcMan_3_GlcNAc_2_ precursor of complex *N*-glycans (109). Conversely, the action of GnT-III on the GnT-I product before α-mannosidase II directs the pathway to hybrid structures (110). Inhibition of α-mannosidase II by 1-deoxymannojirimycin (**11**; Scheme 1) generates “high mannose” type of *N*-glycans. As the pathway progresses through the Golgi, the core GlcNAcMan_3_GlcNAc_2_ can be further modified by a series of GnTs (GnT-II, IV, V, and VI) that substitute a distinct position of the trimannosyl core generating different branches of complex *N*-glycans (Figure 2) (109).

GnT-V expression is increased during oncogenic transformation (111–113), consequently, cancer cells commonly show increased β1-6G1cNAc-branching at the trimannosyl core of N-linked carbohydrates (55, 56, 58–61, 65–67, 114). GnT-III has been found to play an important role in the suppression of metastasis (115–121) as the introduction of bisecting GlcNAc suppresses β1-6 GlcNAc branching formation catalyzed by GnT-V. In the same line, GnT-I knockdown suppresses *N*-glycan branching and the invasive phenotype in HeLa and PC-3-Yellow cells (122).

GnT-V expression correlates with increased cell migration (77, 123, 124) and sensitivity to acute stimulation of growth factors (73). The latter effect is most probably due to the β1-6G1cNAc-branching increasing galectin binding and retention of growth receptors on the cell surface (75, 124, 125). Furthermore, during the development of the malignant phenotype, E-cadherin expression is accompanied by an increase in its β1-6 branched *N*-glycan structures (116, 126). Pinho et al. (116) demonstrated the importance of the *N*-glycan structures of E-cadherin in the development of gastric tumors. The authors demonstrated that modifications in *N*-glycan chains of E-cadherin by GnT-III lends stability to the protein in the cell membrane, contributing to the formation of stable adherent junctions. On the other hand, modifications catalyzed by GnT-V promotes the destabilization of E-cadherin, formation of unstable adherent junctions and inhibition of cell–cell interactions, which promotes gastric tumor invasiveness (116).

Despite the key role GnT-V plays in cancer progression, no inhibitors have been described thus far. However, a very well-known inhibitor of the production of complex β1,6-branched N-linked glycans is the alkaloid swainsonine (**12**; Scheme 1), a transition-state inhibitor of α-mannosidase II that removes α-1,3 and α-1,6 Man residues from the GlcNAc-Man5GlcNAc2-peptide, causing the formation of hybrid structures (127). Inhibition of N-linked oligosaccharide processing by swainsonine in tumor cells has generated attention in its use as an anticancer agent, since reports indicate that this drug inhibits tumor growth and metastasis, augments natural killer and macrophage-mediated tumor cell killing, and stimulates bone marrow cell proliferation (93, 128–130). This drug effect was also associated with the enhanced expression of the metalloproteinases gene (131). Recently, it was demonstrated that swainsonine inhibited cancer cell growth through the activation of the mitochondria-mediated caspase-dependent pathway (132).

Elongation of hybrid and complex *N*-glycans occurs by the addition of a β-Gal to the β1-2 linked GlcNAc yielding the Galβ1-4GlcNAc (LacNAc) moiety, which can be further elongated by the sequential addition of β1-3-linked GlcNAc and β1-4 linked Gal resulting in a poly-LacNAc extention. In addition, Gal can be substituted by a GalNAc forming the GalNAcβ1-4GlcNAc (LacdiNAc) sequence.

*N*-Glycans can be further decorated by the action of a number of transferases that add Gal, Fuc, sialic acid, and sulfate to the antennae (see below) resulting in the mature structure on the nascent protein (82). Those enzymes involved in *N*-glycans elongation and decoration steps act on *O*-glycans and glycolipids as well, so they are described below in the text.

The GlcNAc adjacent to Asn in the core can be modified by the action of α1-6-fucosyltransferase (FucT-VIII) (133). The FucT-VIII is overexpressed in several types of tumors as colorectal, hepatoma, ovarian, lung, and thyroid (134–137) cancer. Muinelo-Romay et al. showed higher activity and increase of FucT-VIII expression in human colorectal tissues correlating with the degree of infiltration through the intestinal and malignant transformation (134). FucT-VIII is also upregulated in non-small cell lung, correlating with tumor metastasis, disease recurrence, and poor survival of patients (137). These correlations have prompted many groups to pursue inhibitors of FucT-VIII as potential antitumorals. Inhibitors of a recombinant α1-6FucT from *Rhizobium sp*. have been described. Several racemic polyhydroxylated indolizidines have been tested as potential inhibitors of this enzyme. One of the castanopermine stereoisomers was the best inhibitor with an IC_50_ of 0.045 mM. Interestingly, this compound turned out to be the best mimic for the structural features of the fucose moiety in the presumed transition state (138, 139).

## *O*-Glycosylation in Cancer

*O*-Glycosylation is a common type of PTM that consists of the attachment of a αGalNAc on Thr or Ser residues from specific sequences of target proteins. The newborn *O*-GalNAc glycan can be further modified by several glycosyltransferases acting in a sequential manner in order to extend the glycan chain either branched or linearly according to substrate specificity. *O*-Glycans are abundantly found attached on the cell surface and on the ECM proteins, especially in the form of mucins, heavily O-glycosylated proteins in which carbohydrate amount can account for 50% of the molecule by weight (140). The biological function of mucins and mucin-like glycoproteins is deeply dependent on its carbohydrate chains (141). Since the synthesis of *O*-glycans is controlled by the availability of substrates and enzymes in subcellular compartments, without any correcting mechanisms, such as N-glycosylation and protein folding, it is easy to understand why aberrant *O*-glycans are usually observed in tumorigenesis and metastasis. Altered O-glycosylation is a universal feature of cancer cells, but only specific glycan changes are frequently associated with tumors. The specific *O*-glycan changes commonly found in cancer cells, as well as its biosynthesis and potential as a drug target are depicted in the following items.

**Figure 3:**
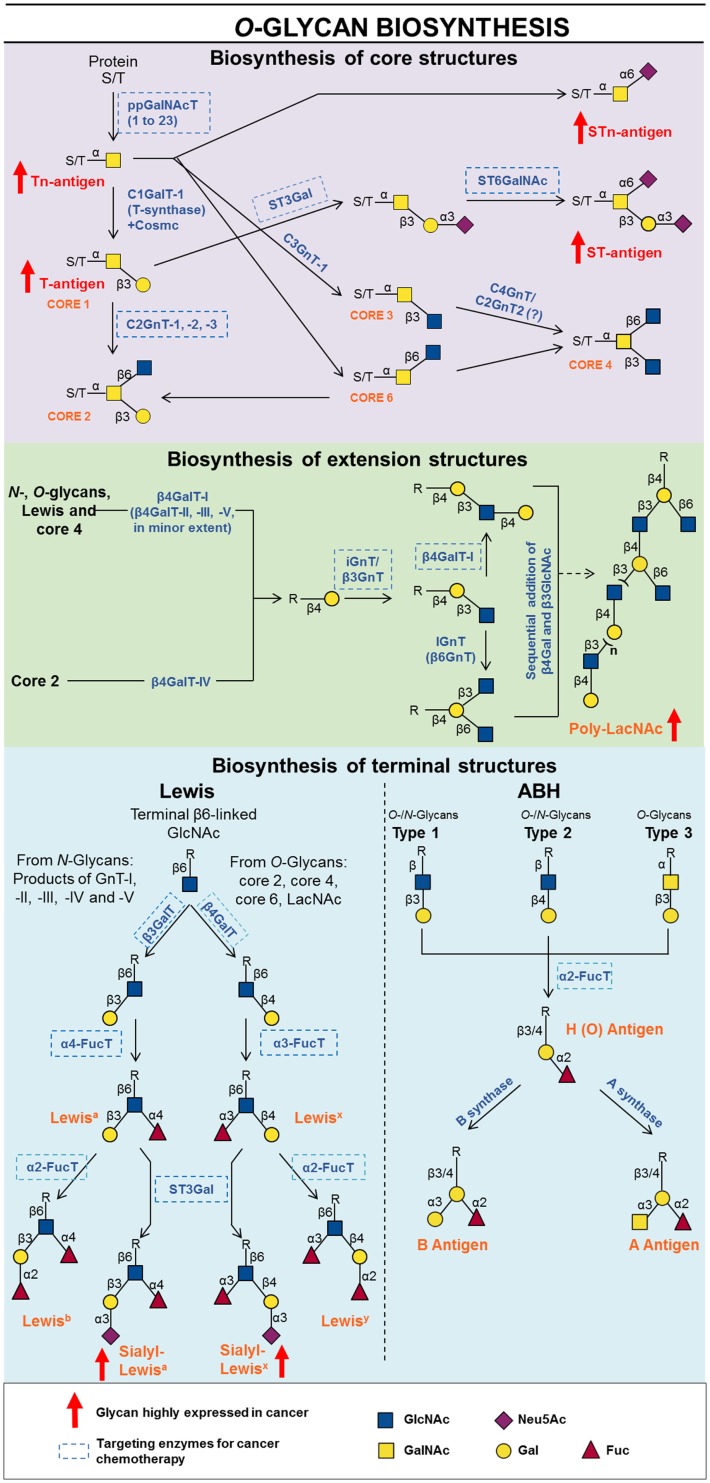
**Schematic representation of biosynthesis and processing of O-linked oligosaccharides showing known inhibitors and key targets for inhibition**.

In cancer, truncated glycan mucin related tumor-associated carbohydrate antigens (TACA), are abnormally expressed in several epithelial cancers (i.e., gastric, pancreatic, colorectal, ovarian, and breast cancers) (142–144). In many types of cancers, enzymes from poly-peptidyl-αGalNAc transferase (ppGalNAcT) family are reported to be located throughout the Golgi, instead of being restricted to *cis*-Golgi as in normal conditions. The ppGalNAcT catalyzes the transfer of a α-GalNAc from UDP-GalNAc to a Ser or Thr residue of a glycoprotein, producing the Tn antigen. When the Tn antigen is generated, it can have three different fates: (i) it can be sialylated on C6 by a ST6GalNAcT; (ii) it can be substituted on C3 or C6 by a β-GlcNAc which gives rise to core 3 or core 6, respectively; or (iii) it can be galactosylated on C3 by the C1GalT1 in order to form core 1, also known as Thomsen-Friedenreich (TF) or T antigen. The emergence of Tn antigen can be a consequence of some abnormality with ppGalNAc-T, C1GalT1 (also known as T-synthase), or with a chaperone responsible for its proper folding or an imbalance in the ratio of the competing downstream core 1 processing enzymes (Figure 3) (145, 146).

Several enzymes from the T-synthase family are reported to be involved in many cancers in both positive and negative fashion. For example, ppGalNAc-T2 is overexpressed in gastric cancer and glioma cells, which is reported to decrease invasion and metastasis (147, 148), while ppGalNAc-T12 is considered to be a negative marker for gastric and colorectal cancer metastasis (149). On the other hand, other ppGalNAc-Ts are shown to be bad prognosis for tumor progression. Bladder cancer tissues contain high levels of ppGalNAc-T1 mRNA and silencing this gene significantly inhibits tumor growth *in vivo* (150). High expression of ppGalNAc-T3 is found in high grade tumors and correlates to poor prognosis in renal carcinomas, gastric carcinomas (151, 152) and in pancreas adenocarcinoma (153). The ppGalNAc-T6 is upregulated in the vast majority of breast cancers and it is suggested to disrupt mammalian cell carcinogenesis (154, 155). The ppGalNAc-T7 has been shown to play important roles in cervical cancer pathogenesis (156) and enhances metastatic process of melanoma cells (157). The ppGalNAc-T13 gene was upregulated in highly metastatic lung cancers (158). The ppGalNAc-T14 has been reported to contribute to ovarian cancer carcinogenesis through aberrant glycosylation of MUC13 (159) and may be a potential biomarker for breast cancer (160). Therefore, the ppGalNAcTs have been proposed as tumor diagnosis and prognosis markers, as well as foranti-cancer immunotherapy. In particular, ppGalNAc-T3 was shown to be recognized as an antigen by cytotoxic T lymphocytes from patients with brain tumor (161), while ppGalNAc-T6 is a marker for the prognosis of pancreatic cancer (162) and breast cancer metastasis (163). Besides the use of ppGalNAcTs as tools for diagnosis and prognosis, this class of enzymes is a potential target for anti-cancer chemotherapy.

Primary attempts to perform kinetic analysis of ppGalNAcT yielded a peptide inhibitor. The EPO-G (**13**; Scheme 1) consists of a 12-residue peptide, which differs from EPO-T, a commercial peptide usually utilized as an acceptor substrate of the enzyme by a single residue. Although EPO-G was observed to inhibit ppGalNAc and helped to define its mechanism of enzyme action, it presented an inexpressive *K*_i_ of 0.77 mM (164).

Another strategy to inhibit ppGalNAc, and consequently whole O-glycosylation, is to use derivatives from UDP-GalNAc. Hatanaka and coworkers had proposed UDP-GalNAc-based inhibitors using the model of tunicamycin, a well-known inhibitor of N-glycosylation. The authors had synthesized compounds substituting UMP with different length fatty acid chains (**14**; Scheme 1), which resulted in the 16 carbons-substituted UMP derivatives having a slight inhibition (IC_50_ of 0.63), of ppGalNAcT (165). Aryl-glycosides of GalNAc (166) were found to competitively inhibit the elongation of *O*-GalNAc chains and have been extensively used for investigating the role of mucin chemical composition on its biological functions and its biosynthesis (167). The derivative benzyl-αGalNAc (**15**; Scheme 1) was shown to decrease the level of sialylation and sulfation of mucins secreted by the human colon carcinoma cell line HT-29 MTX (168), and also decreased the binding of treated HM7 colon cancer cells to E-selectin [ELAM-1, recognizes sialyl Le^a^ and sialyl Le^x^ (169)], which would be a desirable effect in modifying the immunological and biological properties of colon cancer cells. However, the use of benzyl-αGalNAc as a drug is unlikely, because the desired effect is only reached in cell culture with a millimolar range. Such a compound also behaves as a competitive inhibitor of C1GalT1 (166), causing treated cells to express mucins with a high level of the cancer-associated Tn (168, 169).

Synthesis of C-glycosidic UDP-GalNAc mimics (**16**; Scheme 1) were reported, but to our knowledge, their biological activity were not tested (170). *In vitro* evaluation of the 3-, 4-, and 6-methylated UDP-GalNAc compounds (**17**; Scheme 1) effect had shown an inhibition pattern similar to UDP-GlcNAc (171).

Promising compounds targeted to ppGalNAcTs came from the work of Hang and coworkers (172), by screening an uridine-based library designed to target enzymes that utilize UDP-sugar substrates (173). Through this approach, compound **18** (Scheme 1) inhibited a series of ppGalNAcTs (ppGalNAcT-1 to T5, T7, T10, and T11) in micromolar range, suggesting selectivity against this enzyme family, since inverting and retaining GalTs or other UDP-sugar utilizing enzymes were not significantly inhibited. In addition, these compounds inhibited O-glycosylation but not N-glycosylation and induced apoptosis in two different cell types (Jurkat, a lymphoma cell line; and HEK293T). Therefore, compound **18** (Scheme 1) is a promising scaffold for *O*-glycan inhibition in cancer cells.

Recently, Pouilly and coworkers have employed metabolic engineering in order to label highly active metabolic cancer cells with UDP-αGalNAc analogs (**19**; Scheme 1). They observed that all compounds could be recognized by ppGalNAcT1, and that peracetylated GalNAc analogs with hydrophilic substitutions on the *N*-acetyl group, such as azidoacetyl and glycolyl, could be incorporated into cell surface glycoproteins at only slightly lower levels compared to the natural GalNAc. In addition, when mice were immunized with glycopeptides carrying the GalNAc analogs linked to Ser or Thr, some of them had produced an antiserum, which was specifically directed against GalNAzaSer/Thr, without cross-reactivity toward GalNAcSer/Thr. Such a result brings to light that this approach could be used in *O*-glycan biosynthesis research and also for passive immunotherapy of cancer directed at cell surface tumor-associated *O*-glycans (174).

The epitope *O*-GalNAc can be further sialylated on C6 by a ST6GalNAcT (175–178). The disaccharide STn is overexpressed in pancreas, gastric, bladder, breast, and ovarian cancer (142, 179–182). The appearance of STn is explained by the abnormal expression of ST6GalNAc together with its aberrant localization throughout the Golgi apparatus, contributing to the overpass of complex *O*-glycan processing enzymes resulting with expression of short STn-modified *O*-glycans (176). Overexpression of this sialylated antigen reduces cell–cell interactions and increases cell migration (183) most likely by altering cell adhesion to ECM (180, 184). Positive correlations of STn antigen expression with cancer aggressiveness and poor prognosis of the patients has provoked great interest in the functional analyses of STn. In this respect, Ozaki et al. (185) demonstrated that MUC1 carries a large proportion of STn in human advanced gastric cancer tissues.

ST6GalNAcT can also modify core 1 structures and its sialylated derivative comprises the sialyl T (ST) antigen (see below). Such a feature is found in breast cancer due to the increased expression of ST3GalT-1 combined with the low expression or absence of C2GnT-1 (186).

### Core 1, T-synthase, and comsc

The peptide carrying O-linked αGalNAc can be converted to core 1 (Galβ1-3GalNAcα-S/T) structure by core 1 β1-3 galactosyltransferase, termed T-synthase or C1GalT. Cancer cells exhibit a high level of mucins with T and ST antigen (187). T disaccharide represents a cancer-associated antigen in colorectal carcinoma and it is suggested to act as a prognostic marker (188). T antigen is associated with adhesion and metastasis of human breast cancer cells through the binding to galectin-3 (189). Moreover, the overexpression of *MUC1* gene in human and murine mammary carcinoma cells correlates with the increase of T antigen assumedly by the concomitant down regulation of both C2GnT1 and ST3Gal (190), which also causes the loss of adhesive properties to E-selectin of the cells studied, but favors the binding of MUC1-overexpressing cells to galectin-3.

The use of T antigen as a target for anti-cancer immunotherapy is in progress. The T antigen or its difluoro analog were coupled to a glycosynthetic peptide and to a tetanus toxoid (**20**; Scheme 1), affording synthetic vaccines, which induced very strong immune responses in mice overriding the natural tolerance of the immune system. The induced antibodies were selectively directed against the tumor-associated MUC1 structures and strongly bind to breast cancer cells of the MCF-7 cell line (191). This approach can be used for anti-cancer immunotherapy and the difluoro analog of T antigen may serve as an inhibitor for *O*-glycan synthesis. Recently, *Sclerotium rolfsii* lectin was shown to bind to T antigen and inhibit growth of human colon cancer HT29 and DLD-1 cells by binding to cell surface glycans and inducting apoptosis through both the caspase-8 and -9 mediated signaling (192), being an interesting possibility for therapy.

High levels of T antigen have been commonly associated with overexpression of T-synthase (146), or imbalance in the ratio of the competing downstream core 1 processing enzymes, C2GnT and ST6GalNAc-II (145). Remarkably, there is only a single gene encoding T-synthase in humans and other mammals (146), and enzymatic activity of this protein is dependent on its correct folding promoted by a unique chaperone. Cosmc is a molecular chaperone located in ER that promotes correct T-synthase folding and guiding to Golgi (193, 194). Somatic loss-of-function mutations of the chaperone gene Cosmc was shown to abolish the T-synthase activity, leading to the appearance of Tn antigen on the surface of many types of tumor cells, like the highly aggressive fibrosarcoma cell line (195), colon cancer cell line and melanoma-derived cell lines (196). Since Cosmc is encoded by a single gene in the X chromosome, its susceptibility to epigenetic silencing through hypermethylation of its promoter (197) also leads to Tn antigen increase in mucins.

The role of T-synthase in tumors varies according to the type of cancer tissue. Whereas in breast cancer there are multiple reports indicating that T-synthase does not have its expression or activity altered, there are *O*-glycan modifications other than T antigen present, and hepatocellular carcinomas show an overexpression of this enzyme which is associated with poor survival of the affected patients. In addition, the T-synthase plays a role on HGF/MET signaling pathway in hepatocarcinoma cells, leading to a proliferation pattern in this cell type (198). In human colon cancer cells, the suppression of T-synthase by iRNA was shown to be associated with an increased presence of Tn, STn and core 3 glycans in this cells surface, since T-synthase competes with ST6Gal and C3GnT for substrate (199). Based on all the evidence, it would be ideal to either completely or partially inhibit *O*-glycan biosynthesis without altering the core 1 formation.

### Core 2 and C2GnT

Core 2 β1-6 *N*-acetylglucosaminyltransferase (C2GnT) competes with ST3GalNAc for the common core 1 substrate, and the control of mucins decoration with whether ST or core 2-derivated antigens depends on the relative expression of both glycosyltransferases and their subcellular localization. The classification of human C2GnTs were primarily based on the differential enzyme activities observed for C2GnT extracted from leukocytes and mucin-producing cells, known as C2GnT-L and C2GnT-M, respectively. Lately, the human C2GnT variants were classified as -1, -2, and -3, of which C2GnT-1 corresponds to L and C2GnT-2 to M.

Increased levels of C2GnT determine the increased branching of *O*-glycans and it is associated with the acquisition of invasive and metastatic potential rather than simply transformation of cancer cells (200). The expression of C2GnT-1 positively correlates with the progression of prostate cancer in human patients (201), testicular germ cell tumor (202), endometrial carcinoma (203) and with bladder tumor progression (204). In addition, high levels of C2GnT does not necessarily indicate that *O*-glycan chains of mucins will be terminated on core 2, but that there is an increase in the combination of possible glyco-structures, since core 2 is a substrate to further modifications, such as poly-LacNAc, sialic acid, fucose, and sulfate unities, increasing the variety of *O*-glycan chains on the cell surface and expanding the roles for these different glycans. Thus, high levels of C2GnT contributes to increases in core 2 *O*-glycan structures that also serve as substrates to ST3Gal, corroborated by the hypersialylation observed in leukemic leukocytes from chronic myelogenous leukemia and acute myeloblastic leukemia (205). Likewise, core 2 *O*-glycans of MUC1 from bladder tumors are modified with poly-LacNAc, which allows them to bind to galectin-3 and hamper the access of NK cells to the TNF-related apoptosis-inducing ligand present in the tumor cells surface (204). Therefore, core 2 *O*-glycans modified with poly-LacNAc can protect bladder tumor cells from NK cell mediated death, which highly favors metastasis.

On the other hand, C2GnT expression and surface decoration of T cells with *O*-glycans rich in core 2 structures renders them susceptible to galectin-1-induced cell death. It is suggested that this fact may be due to oligosaccharide-mediated clustering of CD45 molecules on the cell membrane, facilitating their binding to galectin-1 (206). Therefore, unlike in other cancer cells aforementioned, C2GnT expression favors T-lymphoma cell death by a particular mechanism and the haploinsufficiency of this enzyme is sufficient for loss of core 2 *O*-glycan expression and galectin-1-induced cell death resistance (207). Likewise, the downregulation of C2GnT-2 was observed in primary tumors from colorectal cancer patients as well as in colon cancer cell lines contrasting with normal colon tissues. The protective role of C2GnT-2 was demonstrated by the suppression of cell invasiveness and tumor growth of C2GnT-2-transfected colon cancer cells *in vitro* and *in vivo* (208).

Inhibiting C2GnT activity is desirable in cancer types in which core 2 is highly expressed and affecting in cancer progression, as well as in tumor cells containing the poly-LacNAc or hypersialylation phenotype, since C2GnT has been shown to act as a key regulator for such modifications (209, 210). However, a C2GnT inhibitor must be carefully designed, because such an enzyme shares homology with many other UDP-GlcNAc:*N*-acetyl-glutaminyl transferases. In addition, the use of UDP-GlcNAc analogs could potentially interfere with biosynthetic pathways involved in the interconversion, metabolism, and transport of these sugar nucleotides (211). For this reason, the first attempt to inhibit C2GnT was made by targeting its acceptor substrate with deoxy analogs (**21**; Scheme 1). However, they displayed a poor inhibitory effect, with a *K*_i_ of 0.56 mM, much higher than the enzyme *K*_m_ (211). Kuhns and coworkers had also used several deoxy-analogs of core 1 to inhibit C2GnTs extracted from a series of tissues. Although they also obtained disappointing results, they at least observed differential binding requirements for the hydroxyl groups in the substrate from the enzymes explored (212). All types of C2GnT seem to have an absolute requirement for the 4- and 6-hydroxyls of GalNAc, and the 6-hydroxyl of Gal in the substrate, but the recognition of the 3- and 4-hydroxyls of galactose and the acetamido group of GalNAc is variable between C2GnT-1 and C2GnT-2. Molecules coupled to the chemically inert, but UV-detectable *p*-nitrophenol (*p*NP) group would serve as photoaffinity labeling reagents. Thus, a *PnP* substituted core 1, Galβ1-3GalNAcα-*p*NP (**22**; Scheme 1), an acceptor substrate analog, became a potent inhibitor upon 350 nm irradiation (213) with little inhibition of other *O*-glycan processing enzymes, even in higher doses.

Despite its potential application in research and clinical practice, there is no recent progress in the design of C2GnT inhibitors, but interesting scaffolds may arise from the studies of UDP-GlcNAc epimerase inhibitors and other UDP-GlcNAc transferases. Meanwhile, inhibitors of core 2 or poly-LacNAc-modified core 2 interactions with galectins are under review and may be another avenue to combat tumor progression promoted by these *O*-glycans.

## Poly-*N*-Acetyllactosamines

*N*-Glycans, core 1, and core 2 *O*-glycans can also be extended by *N*-acetylglucosaminyltransferases and galactosyltransferases to form sequences that represent the little i antigen. Linear poly-LacNAc units can be branched by members of the I-branching β-1,6-*N*-acetylglucosaminyl transferase (IGnT), resulting in the large I antigen. Moreover, poly-LacNAc chains can be synthesized from core 4, which arises from the addition of a β1-6GlcNAc on *O*-GalNAc from core 3 (Figure 3).

The control of chain length depends primarily on β4galactosyltransferase (β4GalT). There are seven β4GalTs characterized to date (214–219), among which β4GalT-I and β4GalT-III variants are the most widely expressed (217). The others are expressed tissue-specifically to a minor extent (217). β4GalT-I was the first isolated galactosyltransferase and it is known to act over a variety of substrates, being responsible for poly-LacNAc synthesis in *N*- and *O*-glycans, Lewis structures and core 4 O-linked carbohydrates. β4GalT-II, -III, and -V can also use these types of substrates but to a lower extent. In turn, β4GalT-IV is the one responsible for galactosylation of core 2 terminus from *O*-glycans (220). Furthermore, β4GalT-IV, but not β4GalT-I, drastically reduces its efficiency as the acceptors become longer (220), consistent with the fact that poly-LacNAcs on core 2 branched oligosaccharides are shorter (only two LacNAc repeats) than those in *N*-glycans (220). β4GalT-VI and β4GalT-VII do not act on *N*- or *O*-glycans, but were shown to synthesize Galβ(1-4)Glcβ-ceramide from Glcβ-ceramide (219) and to participate on proteoglycan biosynthesis (221), respectively.

The β3-*N*-acetylglucosaminyltransferase, which acts over β4Gal, was first characterized and called extension IGnT because it creates a new terminus for β4GalT to act upon (222). Besides IGnT, other β3GnTs were shown to be capable of both initiating and elongating poly-LacNAc chains. β3GnT-1, -2, -3, and -4 enzymes were found to act the same as IGnT, even though they do not share structural similarity with IGnT, but they do share conserved motifs with the β3GalT family (223, 224). These β3GnT enzymes were shown to act preferentially over poly-LacNAc and are suggested to be involved in the biosynthesis of poly-LacNAc sugar chains (224). In addition, their expression profiles were different among human tissues (223, 224).

The IGnT, creates multiple branches on the poly-LacNAc chain, which may serve as a mechanism for amplifying the functional potency of cell surface glycoproteins (225). There are three human isoforms of IGnT characterized thus far (226, 227), from which IGnT-1 and IGnT-2 showed similar tissue expression profiles, with the transcript expression of IGnT2 greater than that of IGnT1 and IGnT-3 (227). It is noteworthy that C2GnT-2 is also considered as an I-branching enzyme, although it exhibits only weak peridistal I-branching activity (228).

Increasing evidence demonstrates the association of poly-LacNAc chains found both in *O-* and *N*-glycans with cancer. Thyroid papillary carcinomas were observed to present high heterogeneity in their poly-LacNAc chain length and branching status, different from those produced in other thyroid neoplasms (229). Poly-LacNAc substituted oligosaccharides were shown to be expressed in a metastasis-dependent manner on melanoma cells (230) and also to be important for the interaction of carcinoma cells with hepatic cells in the process of liver metastasis (231). In addition, poly-LacNAc substitutions on *O*-glycans render prostate cancer cells resistant to NK cell toxicity, cooperating for prostate cancer cells to survive longer in host blood circulation and favoring the metastasis process (232).

Most of the associations between increased expression of poly-LacNAc and cancer occur through the binding of these oligosaccharides to galectins. Such interactions have been shown to mediate lung metastasis of melanoma cells through the adhesion of poly-LacNAc from *N*-glycans on galectin-3 (233) and to be important for tumor immune evasion, as demonstrated by the increase in tumor lymphocytic infiltration and tumor-specific cytotoxic T cells and decrease on melanoma growth *in vivo* after the treatment with a metabolic inhibitor of LacNAc biosynthesis (234).

Given the role of poly-LacNAc in cancer, it would be interesting to inhibit its binding to galectins, which can be achieved in two different manners: by inhibiting the binding itself, through blocking galectins, or by inhibiting enzymes involved in the LacNAc biosynthesis. In fact, poly-LacNAc processing enzymes have been shown to have a direct correlation with cancer.

For example, the overexpression of β4GalT-I, -II, and -V was suggested to cause the increase of galactosylation in human astrocytomas, which is associated with its malignancy (235). Particularly, β4GalT-IV was shown to behave as a strong predictor for tumor metastasis and is associated with poor overall survival of colorectal cancer patients (236). The upregulation of the expression of β4GalT-III in neuroblastoma (NB), correlates with advanced stage, unfavorable histology, and predicts a poor prognosis in NB patients. In addition, β4GalT-III expression increased cell migration, invasion, and tumor growth of NB cells possibly due to modified glycosylation of β1-integrin through increasing terminal Gal (237). Interestingly, there are an increasing number of reports showing that metastatic cells contain elevated levels of β4GalT-I on the cell surface, where it serves as an adhesion molecule (238, 239), whereas the Golgi levels of that protein remains the same between non-metastatic and metastatic cells. Elevated expression of surface β4GalT-I, characteristic of highly metastatic murine melanoma cells, contributes to their invasive phenotype *in vitro* and to their metastatic phenotype *in vivo* (240). The expression of β4GalT-I on the cell surface plays a crucial role in the proliferation of MCF-7 cells through its activity as a membrane receptor (241). In addition, cell surface β4GalT-I is suggested to induce multidrug resistance through the hedgehog pathway in the human leukemic cell line (242). These results reinforce the β4GalT-I as an unexpected target for inhibition due to its role in the carcinogenic process at intra and extracellular levels.

The design of the first β4GalT-I activity inhibitors were guided by the substrate specificity and mutagenesis studies of commercially available bovine β4GalT-I. Phosphonate donor substrate analogs were proposed to be interesting scaffolds since the ­phosphonic group would mimic the biphosphate of UDP and complex with the Mn^+2^ divalent ion present in the binding pocket of the enzyme. Nevertheless, of the synthesized adenosine 5´-­phosphoric α-d-glucopyranosylphosphonic anhydride, guanosine 5´-phosphoric α-d-mannopyranosylphosphonic anhydride, and uridine-5´-phosphoric-α-galactopyranosylphosphonic anhydride (**23**; Scheme 1), only the last one inhibited β4GalT-I, with an apparent *K*_i_ of 165 μM. The compounds carrying hexose isomers other than Gal bound to purines did not display β4GalT-I inhibitory activity but showed slight cytotoxicity against B- and T-lymphoblastic leukemia cells (243), probably by a mechanism independent of β4GalT. The substitution of the diphosphate anhydride group by a hydroxylphosphinylmethylphosphate (**24**; Scheme 1) lowered the *K*_i_ value from 165 to 97 μM, but still did not show any *in vitro* antitumor activity (244). In addition, other molecular groups were substituted to mimic the interaction between the pyrophosphate of UDP and the metal ion of β4GalT active site. The substitution of pyrophosphate by a malonyl group yielded a compound (**25**; Scheme 1) with poor inhibitory activity, whereas the replacement by a Glc was more effective, resulting in a compound (**26**; Scheme 1) with satisfactory inhibitory activity (*K*_i_ = 119.6 μM) (245).

A novel compound was designed based on the model of an SN2-like transition state of two substrates for β4GalT activity. This model has two strategic characteristics: the use of a natural UDP as the leaving group instead of phosphonate and the linking of the acceptor (GlcNAcβ-OMe) and the donor (Gal moiety) via a methylene ether. The resulting tricomponent bisubstrate analog **27** (Scheme 1) showed a remarkably potent inhibitory activity toward bovine β4GalT-I, displaying *K*_i_ values of 1.35 μM for acceptor and 3.3 μM for donor substrate (246).

In an effort to avoid nonspecific binding of UDP-Gal-based inhibitors to other galactosyltransferases, Chung and coworkers developed selective β4GalT inhibitors based on the acceptor substrate. Such compounds had GlcNAc attached to aromatic aglycone moieties (**28**; Scheme 1) and exhibited *K*_i_ of 3.5–9.5 μM. The replacement of the aromatic group to other aglycones resulted in poor inhibition, suggesting that the aromatic ring is responsible for the drastic increase in the binding affinity of inhibitors (247).

Takayama and coworkers have used rapid electrospray mass spectrometry to identify selective inhibitors from a library based on the donor-sugar nucleotide UDP-Gal. From the iminocyclitols and phosphonates screened the compounds (**29**; Scheme 1) and UDP-2-fluoro-Gal (UDP-2-F-Gal, **30**; Scheme 1) were the most effective, displaying 95% and 90% of enzyme inhibition at 1 mM. The UDP-2-fluoro-Gal also exhibits an IC_50_ of 202 μM (248).

The publication of bovine β4GalT-I crystal structures presenting the unbound (249) and the UDP-Gal bound forms (250) was of outstanding importance in demonstrating that a loop containing the Trp314 (equivalent to Trp310 in human β4GalT-I) moves toward the active site upon donor substrate binding. Based on this information, Takaya and coworkers have substituted C2 or C6 of Gal from UDP-Gal with a flexible trioxadecanyl group linked to a terminal naphthyl group in order to enhance the stacking interaction with Trp314 and to block the acceptor substrate entrance. Indeed, modification at the C6 position (**31**; Scheme 1) was more effective than the C2 position (**32**; Scheme 1) of Gal residue, yielding the strongest competitive inhibitor (*K*_i_ of 1.86 μM) against UDP-Gal (*K*_m_ of 4.91 μM) thus far known (251).

Aiming to improve the design of biologically applicable inhibitors of galactosylation, a large series of modifications was made on GlcNAc to optimize the acceptor inhibitor geometry (252). From these studies, Brockhausen and coworkers made several observations that must be taken into account on the design of β4GalT targeted inhibitors. However, the best *K*_i_ values obtained, 0.06 mM for 1-thioGlcNAcβ-(2-naphthyl) (**33**; Scheme 1) and 0.01 mM for 1-thio-*N*-butyrylGlcNAcβ-(2-naphthyl) (**34**; Scheme 1), were almost 10-fold higher than inhibitors reported previously (251). The compound 1-thio-*N*-butyrylGlcNAcβ-(2-naphthyl) (**34**; Scheme 1) was further observed to inhibit 68–95% of human β4GalT activity from a series of tumor cell line lysates, without compromising the activity of other enzymes (253). In addition, the specificity of (**34**; Scheme 1) β4GalT was confirmed by testing the enzyme activity of recombinant glycosyltransferases with and without the compound.

In contrast with galactosyltransferase, the relation of IGnTs or β3GnTs expression in cancer has not been investigated well. Only recently, a new member of IGnT family, β3GnT-VIII, was cloned, characterized, and shown to have its transcript levels significantly increased on colorectal cancer tissues compared to normal tissues (254). The same scenario can be observed for I-branching IGnTs. The pioneering work from Zhang et al. (255) has demonstrated that IGnT-2 is overexpressed in highly metastatic breast cancer cell lines of human and mouse origin and basal-like breast tumor samples. Moreover, IGnT-2 expression was significantly correlated to the metastatic phenotype in breast tumor samples and its ectopic expression enhanced cell detachment, adhesion to endothelial cells, cell migration, and invasion *in vitro* and lung metastasis of breast cancer cells *in vivo*. The knockdown of IGnT-2 resulted in the elimination of metastatic aspects making this enzyme a promising target for metastatic breast cancer (255).

Information on IGnTs and IGnTs inhibitors are more limited than reports about their biological roles. In fact, there are no inhibitors that target extension or branching of *N*-acetylglucosaminyltransferases by acting on *O*-glycans. Features for developing inhibitors were based on studying the characterrization of the isolated and purified enzymes activity, from which β3GnT was observed to be inhibited by the product UDP (~70% inhibition at 1 mM) and by 4-thiouridine diphosphate (256). I-branching β6GnT was also inhibited by UDP and UTP, indicating that the uracil moiety and the number of phosphodiesters appear to be important for the enzyme binding and activity (257). Nonetheless, potent inhibitors can come from tests with UDP-GlcNAc analogs (258), used as inhibitors of others *N*-acetylglucosaminyltransferases, or from acceptor substrate analogs, which provide potent scaffolds for *N*-glycan branching GnT-V inhibition.

## Lewis and ABH Blood Group Antigens

Lewis and ABH antigens are terminal oligosaccharide structures that can be attached to βGal, found in O- and N-linked glycans and glycolipids (Figure 3). They are termed “blood group antigens” because they were initially discovered on red cells, although they were afterward observed on the surface of many other cell types, such as epithelial and endothelial cells (259).

There are three main types of disaccharide precursors of ABH and Lewis antigens found in glycoproteins: (i) type 1, Galβl-3GlcNAcβ; (ii) type 2, Galβl-4GlcNAcβ; and (iii) type 3, Galβl-3GalNAcα. Type 1 and type 2 are found in lacto series of glycolipids and in *N*- and *O*-glycans. In *O*-glycans, type 1 originated by the activity of β3-galactosyltransferase 5 (β3GalT-5), the unique isoform known in humans, and type 2 consists of terminal LacNAc moieties, synthesized by a set of β4-GalTs as already mentioned in the previous section. The disaccharide of type 3 represents the core 1 or T antigen of *O*-glycans and is found exclusively in these structures (Figure 3) (260).

The H antigen is generated by α1-2 fucosyltransferases (α1-2FucT), encoded in humans by *FUT1* and *FUT2* genes, which adds an αFuc on terminal βGal residues of the 3 types of disaccharide precursors. The A and B antigens are originate from the same H precursor by the action of A and B enzymes that branch from the Galβ at C3 by adding an GalNAcα or Galα residue, respectively (261).

The increase of H-antigen correlates with the loss of A- and B-transferases expression and activity in red blood cells at the preleukemic stage (262), bladder malignant urothelium cells (263), and oral epithelia (264). The loss of blood group antigen A expression has a negative prognostic impact in stage I non-small cell lung cancer, especially in patients with adenocarcinoma (265). The increase in H epitope in cancer cells is related to α1-2FucT overexpression. Upregulated expression of *FUT1* and β-*N*-acetylgalactosaminyltransferase and prostate-specific antigen (PSA) levels are biomarkers for prostatic cancer (266, 267). Moreover, 1,2-fucosylated glycans, at the surface of rat colon carcinoma cells, were associated with increased tumorigenicity, resistance to natural killer cytotoxicity and apoptosis (268, 269). Besides, a glucose analog of H-antigen, the 2-fucosyl lactose (H-2g), stimulates angiogenesis in endothelial cells (270).

Lewis antigens can be derivated only from type 1 and 2 precursors, by the addition of a αFuc to the position 4 or 3 of GlcNAc, giving the Le^a^ or Le^x^ antigens, respectively. Addition of other Fuc in the same position of the H types 1 and 2 antigens give the Le^b^ and Le^y^ antigens, respectively. The difucosylated Le^b^ and Le^y^ can also be synthesized from A and B antigens by the action of the same *N*-acetylglucosaminide: 3/4-αfucosyltransferases (α1-3/α1-4FucT) that participate in Le^a^ and Le^x^ biosynthesis (Figure 3) (271).

Cancers that are known to express Le^Y^ include ovarian, pancreatic, esophageal, stomach, colon, rectal, and lung cancers (272–276). Because the expression of Le^Y^ in normal tissues is low and it is highly expressed in cancer cells it is a good potential therapeutic target (276, 277). In fact, the use of Le^Y^ in cancer vaccines (278, 279) and immunoconjugated chemotherapy (280) are in progress. In addition, Le^Y^, together with *FUT1* gene upregulation, is suggested to be involved in cell migration required for the early steps of tumor angiogenesis (281). Yan et al. (272) found that overexpression of α1-2FucT in ovarian cancer cell line correlates with overexpression of Le^Y^, increased invasiveness and poor prognosis for such types of cancer. *FUT1* also modulates cell proliferation in the HER2-positive cancer cell line NCI-N87. Authors suggest that knockdown of *FUT1* down regulates HER2 signaling via EGFR down regulation (282). Thus, *FUT1* may serve as a new molecular target for HER2-overexpressing human cancers with activated EGFR signaling. Consequently, to inhibiting α1-2FucT-mediated cancer processes is an important matter, which has been the aim of several researchers. Palcic et al. (283) designed the first bisubstrate analog inhibitor (**35**; Scheme 1), containing structural elements of both, donor and acceptor of α1-2FucTs. The analog of the postulated transition-state was a potent inhibitor of porcine submaxillary α1-2FucTs in cell free systems, *K*_i_ = 2.3 μM. The same group demonstrated the inhibition of porcine submaxillary α1-2FucTs by deoxygenated oligosaccharide acceptor analogs (**36**; Scheme 1) with a *K*_i_ = 0.80 mM, but was not tested in cell systems (211).

The sialylated variants of Le^a^ and Le^x^, sialyl-Le^a^ (SLe^a^) and sialyl-Le^x^ (SLe^x^), can be synthetized by the addition of a sialic acid in α2-3 linkage either onto Le^a^ or Le^x^ antigens or onto type 1 and type 2 precursors followed by its fucosylation on C3 or C4 of the GlcNAc moiety (Figure 3). Cancer cells have the ability to mimic immune cells, where they migrate to inflamed sites by expressing SLe^a^ and sialyl SLe^x^ on its surface which allows them to attach onto E- and P-selectins expressed by the endothelium (284, 285). Such sialylated oligosaccharides may also mediate metastasis by forming cellular thrombus through the binding to platelet-borne P-selectins, which can block leukocyte infiltration into tumors (285).

The expression of SLe^a^ was shown to be useful as a marker for colorectal carcinoma aggressiveness and prognosis (286) and is associated with metastasis of pancreas carcinoma cells (287), lung cancer cells, and liver cancer cells by the property of the saccharide to adhere to ELAM-1 of the endothelium (288). Overexpression of SLe^x^ correlates to tumor aggressiveness and prognosis in another set of cancer cells, consisting of prostate (289), colorectal (290), liver, and lung cancers (288, 291). In some types of tumors, as in breast cancer and melanoma, the appearance of both SLe^a^ and SLe^x^ is associated with the tumor emergence and higher expression of these epitopes correlates to the degree of malignancy (292, 293).

The increased expression of SLe^x^ and SLe^a^ antigens on tumor cells could be due to the upregulation of the genes encoding the enzymes responsible for the saccharide biosynthesis, or due to deficiency in the enzymes responsible for sulfation, which normally lead to the generation of Sialyl 6-Sulfo Lewis^x^ antigen and α2-6-sialylation resulting in the production of disialyl Le^a^, present on the normal epithelium (294). In fact, mRNA levels of α1-3/1-4 FucT and ST3Gal are shown to be higher in colon adenocarcinoma cells (295). Therefore, the enzymes that participate on the biosynthesis of SLe antigens are targets for drug design.

There are six sialyltransferases reported to act upon C3 from terminal βGal residues, called ST3Gal-I to VI. Like α1-3/1-4 FucT, sialyltransferases also exhibits preferences toward substrates: ST3Gal-I and -II have a clear preference for the type 3. ST3Gal-III works on types 1 and 2 with a preference for type 1, whereas the opposite is observed for ST3Gal-IV. ST3Gal-V uses lactosylceramide as a substrate to generate the glycolipid GM3, while ST3Gal-VI works on type 2 precursors exclusively (296–298).

ST3Gal III is directly implicated in the enhancement of surface SLe^x^ levels in pancreatic ductal adenocarcinoma and plays a key role in several steps of tumor progression such as E-selectin adhesion, migration and metastasis formation (299). Increased expression of ST3Gal III in pancreatic ductal adenocarcinoma is concurrent with the increase of ST3Gal IV, suggesting their involvement in this pathology is probably due to the promotion of SLe^x^ or SLe^a^ biosynthesis (144).

Sialyltransferases are an evident target for drug design strategies and their expression can be a useful prognostic marker of malignant disease. In 1975, Bernacki (300) indicated that CMP inhibits the transfer of Neu5Ac from CMP-Neu5Ac to appropriate acceptor substrates by using rat liver microsommes as the source of sialyltransferase. From this finding, several donor, transition-states, bisubstrate, and acceptor analogs based on CMP-Neu5Ac have been synthesized and evaluated for inhibitory activity against sialyltransferase (301, 302). Schmidt and colleagues have extensively studied inhibitors of sialyltransferase based on CMP-Neu5Ac and its oxocarbenium transition state in ST-catalyzed reactions. They revealed that R-hydroxyphosphonate esters of CMP (**37**; Scheme 1), with a flattened ring system, are very strong sialyltransferase inhibitors, having *K*_i_ values in the nanomolar range (303). Concerning the disaccharide nucleoside, two compounds, inhibited sialyltransferase on the lymphocyte surface, which resulted in the decrease incorporation of sialic acid into endogenous cellular acceptors or into exogenous desialylated glycoconjugates (304). Sialyltransferase inhibition by the sialic acid-nucleoside analog (**38**; Scheme 1) (301) in a colon adenocarcinoma mouse model resulted in a significant prevention of lung metastasis and prolonged the survival.

Non-substrate-like inhibitors have also been published as inhibitors of sialyltransferase activity. A lithocholic acid analog, Lith-O-Asp (**39**; Scheme 1), inhibited the sialylation of integrin-β1. In addition, Lith-O-Asp altered protein expression levels and the phosphorylation state of various proteins involved in crucial metastasis and angiogenesis pathways, such as vimentin and RNH1, which inhibited angiogenesis and tumor growth *in vivo*, through angiogenin inhibition (305). Soyasaponin I (**40**; Scheme 1), a natural compound purified from soybean, has been shown to be a ST3Gal I inhibitor (306). It effectively inhibited breast cancer cells and murine melanoma cells from metastasis *in vivo* (307, 308). Since, soyasaponin I is difficult to obtain amounts large enough for cancer treatment, a series of lithocholic acid analogs derived from soyasaponin I were synthesized (**41**; Scheme 1) (309). The compound AL10 (**42**; Scheme 1), an ST3Gal and ST6Gal inhibitor, inhibited cell migration and invasion *in vitro* and suppressed lung metastasis in animal studies (310).

Whereas ST3Gal is the most obvious target among the enzymes that participate in Lewis biosynthesis, there is evidence that the inhibition of glycosyltransferases downstream can be an alternative for chemotherapy focused on Lewis glycans. The suppression of β3GalT5 gene was shown to reduce SLe^a^ expression in pancreas adenocarcinoma cell line (311). Other studies have correlated the expression of β3GalT5 with cancer epitopes, such as extended type 1 chains on lactosylceramides of human colon carcinoma (312) and CA19.9 in human pancreatic cancer tissue (313). Recently, Gao and coworkers have reached almost 100% inhibition of β3GalT5 activity with bivalent imizadolium salts (**43**; Scheme 1), nevertheless, these compounds presented little specificity against the enzyme (314). Conversely, the variety and number of inhibitors targeted to α1-3/1-4FucT is far greater than those targeted to β3GalT considering the extensive correlation between those enzymes and cancer progression.

Currently, the products of six *FUT* genes able to catalyze the addition of a Fuc residue in α1-3 or α1-4 position are known in humans. The *FUT3* and *FUT5* enzymes can use both type 1 and 2 precursors as substrates; *FUT3* has a marked preference for type 1 and *FUT5* for type 2. The *FUT4*, *FUT6*, *FUT7*, and *FUT9* enzymes catalyze the addition of a Fuc exclusively onto type 2 precursor. In addition, *FUT3*, *FUT4*, *FUT5*, and *FUT6* are able to fucosylate internal LacNAc motifs from poly-LacNAc structures (315), giving rise to more complex structures like dimeric Le^x^ or trimeric Le^y^ that can be further modified.

For instance, α1-3/1-4FucT protein expression was associated with poor prognosis in various types of cancer (316) and increased E-selectin adhesion and metastatic potential of human lung adenocarcinoma cells (317, 318). Gene knockdown approaches in leukemia T cell that decreased cell adhesion to E-selectin, reinforced this correlation (319). Besides, downregulation of FucT-3 and -5 through shRNA decreased levels of Lewis antigens, adhesion and binding capacities of gastric cells MKN45 (320). Carvalho et al. (321) revealed an increased expression of SLe, *FUT5* and *FUT6* during cell confluence of MKN45, which associated these enzymes with tumor progression. Guo et al. found that *FUT6* was capable in promoting hepatocellular cell growth *in vivo* and *in vitro*, by modulating P13K, and also suggesting *FUT6* as a promising biomarker and a potential therapeutic target for hepatocellular carcinoma (322). Moreover, another study showed that *FUT6* was also increased in prostate cancer. A significant reduction of bone metastasis in a *FUT6*-induced bone metastasis mouse model of prostate cancer was achieved by using a Fuc mimetic inhibitor 2-F-peracetyl-Fuc to inhibit fucosyltransferase enzyme activity (323). In a recent study, Okeley and coworkers demonstrated that oral treatment with 2-F-Fuc provided complete protection from tumor engraftment in a syngeneic tumor vaccine model. The compound inhibited neutrophil extravasation, and delayed the outgrowth of tumor xenografts in immune-deficient mice (324). Zandberg and colleagues revealed that the mimetic inhibitor, 5T-Fuc (a peracetyletaed 5-thio-Fuc, **44**; Scheme 1), blocked the α1-3/1-4FucT activity and decreased SLe^x^ on HepG2 cells. Furthermore, the group demonstrated that treatment with 5T-Fuc impaired selectin-mediated cell adhesion (325). The non-substrate related Panosialins A and B (**45**; Scheme 1) were used as an inhibitor of *FUT7* and suppressed the expression of selectin ligands, on human leukemic lymphoma cell line (U937), which inhibits selectin-mediated cell adhesion (326). On the same cell line, a series of peracetylated *N*-acetyllactosamine (LacNAc) analogs (**46**; Scheme 1) exhibited an enhanced affinity by FucT- VI caused by a 90% inhibition of SLe^x^ expression (327).

Donor substrate mimetic compounds were widely explored as inhibitors of human FucTs. Unnatural sugar nucleotides, UDP-Fuc, ADP-Fuc and CDP-Fuc were tested against FucT-III. Unexpectedly, the enzyme does not only tolerate the exchange of guanosine for adenine but may also accept a pyrimidine base. UDP-Fuc and CDP-Fuc were utilized with lower efficiency than UDP-Fuc, nevertheless, they could act as Fuc donors. A series of GDP-Fuc derivatives was synthesized, purified and characterized in detail for their inhibition kinetics. Compound **47** (Scheme 1) had a *K*_i_ of 29 nM for human FucT-VI (328). Addition of hydrophobic moieties to the Fuc C6 seems to yield potent inhibitors. This strategy was explored to identify selective inhibitors for human recombinant FucT-V. It has been shown that both GDP-2F-Fuc and GDP-6F-Fuc (**48**; Scheme 1) act as competitive inhibitors of *FUTs 3*, *5*, *6*, and *7* with *K*_i_ values in the low micromolar range (329). Peracetylation of GDP-2F-Fuc improved its cell permeability and dramatically reduced cell surface fucosylation. Treatment of human HL-60 cells with the permethylated GDP-2F-Fuc nearly abolished synthesis of Le^X^ and SLe^X^ and led to significant decreases in E- and P-selectin binding (330).

Several *C*-glycosides were synthesized as potential inhibitors of FucT (331). Among the compounds tested, the αMan*p*1-3CH2GalNAc (**49**; Scheme 1) displayed a mixed inhibition of FucT-VI, with respect to both the donor sugar GDP-Fuc and the acceptor LacNAc (332). However, a *C*-glycosyl ethylphosphonophosphate analog of GDP-Fuc (**50**; Scheme 1) presented only a weak inhibition against *FUT3* (IC_50_ value of 2 mM). The modest activity was attributed to the α-anomeric configuration of this C-glycosyl analog, which is opposite to the β-configuration of the natural donor substrate GDP-Fuc (333).

Many other molecules synthesized have inhibitory activity: gallic acid (**51**; Scheme 1) and its derivates were identified as FucT-VII inhibitor (IC_50_ of 60 nM) (334); stachybotrydial (**52**; Scheme 1), isolated from the culture broth of the fungus *Stachybotrys cylindrospora*, were identified to be a potent FucT-V inhibitor (*K*_i_ of 10.7 μM) (335). Together these results point to the potential therapeutic applications for molecules that selectively block the endogenous generation of fucosylated glycan structures.

## Conclusion

The potential of glycans as tools in cancer diagnosis and prognosis is unquestionable. This overview presents the importance of glycan inhibitors as possible anti-cancer drug targets. Several hundred targets exist for the development of inhibitors. The repertoire of available compounds, although extensive, contains few agents that have the affinity and specificity required for converting a laboratory reagent into a drug. However, the few drugs that have been developed have already proven their value as therapeutic agents. These success stories only represent the beginning of the importance of the glycobiology field to anti-cancer chemotherapy.

## Conflict of Interest Statement

The authors declare that the research was conducted in the absence of any commercial or financial relationships that could be construed as a potential conflict of interest.

## Supplementary Material

The Supplementary Material for this article can be found online at http://journal.frontiersin.org/article/10.3389/fonc.2015.00138

Scheme 1 **Inhibitors of enzymes involved in the biosynthesis of glycans derived from the HBP**.
